# Marmoset monkeys use different avoidance strategies to cope with ambient noise during vocal behavior

**DOI:** 10.1016/j.isci.2023.106219

**Published:** 2023-02-16

**Authors:** Julia Löschner, Thomas Pomberger, Steffen R. Hage

**Affiliations:** 1 Neurobiology of Social Communication, Department of Otolaryngology – Head and Neck Surgery, Hearing Research Center, University of Tübingen, Medical Center, Elfriede-Aulhorn-Strasse 5, 72076 Tübingen, Germany; 2 Werner Reichardt Centre for Integrative Neuroscience, University of Tübingen, Otfried-Müller-Str. 25, 72076 Tübingen, Germany; 3 Graduate School of Neural & Behavioural Sciences - International Max Planck Research School, University of Tübingen, Österberg-Str. 3, 72074 Tübingen, Germany

**Keywords:** Biological sciences, Behavioral neuroscience, Machine learning

## Abstract

Multiple strategies have evolved to compensate for masking noise, leading to changes in call features. One call adjustment is the Lombard effect, an increase in call amplitude in response to noise. Another strategy involves call production in periods where noise is absent. While mechanisms underlying vocal adjustments have been well studied, mechanisms underlying noise avoidance strategies remain largely unclear. We systematically perturbed ongoing phee calls of marmosets to investigate noise avoidance strategies. Marmosets canceled their calls after noise onset and produced longer calls after noise-phases ended. Additionally, the number of uttered syllables decreased during noise perturbation. This behavior persisted beyond the noise-phase. Using machine learning techniques, we found that a fraction of single phees were initially planned as double phees and became interrupted after the first syllable. Our findings indicate that marmosets use different noise avoidance strategies and suggest vocal flexibility at different complexity levels in the marmoset brain.

## Highlights

• Monkeys are able to cancel their calls to avoid interfering ambient noise
• Animals seem to find it easier to cancel calls toward their expected end
• Marmosets change their vocal behavior in reflexive and adaptive ways
• We found indications of active noise-dependent interruption of call sequences

## Graphical abstract

**Figure:**
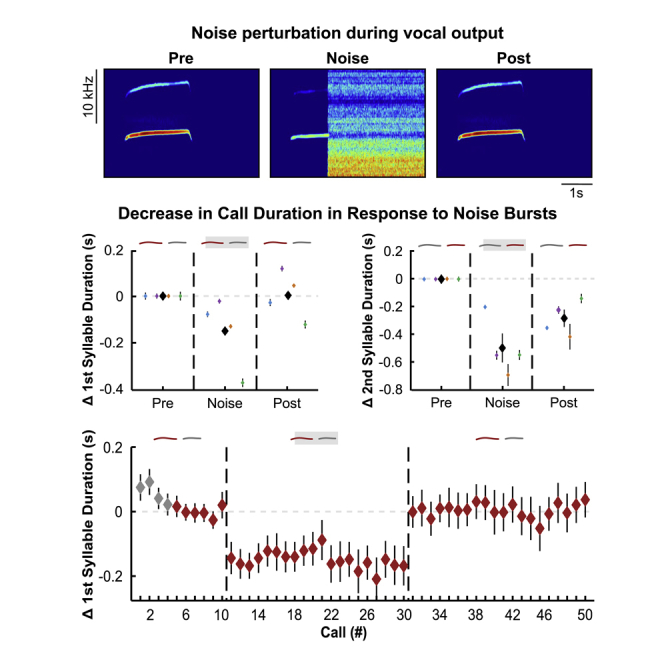

## Introduction

Communication between animals is a crucial trait for evolutionary success.^1^ In many bird and mammalian species, vocal signals have evolved as one of the dominant forms of direct communication between individuals.^2^^,^^3^^,^^4^ These vocal signals are usually produced within noisy environments; therefore, several mechanisms have evolved to compensate for masking acoustic interference, such as heavy rain, wind, and animal or urban sounds.^5^

The mechanisms used to deal with acoustic disturbances can generally be divided into two main types. The first type is modulation of vocalizations that are directly confronted with an increase in ambient noise, i.e., when they are produced during acoustic perturbation. Here, the Lombard effect, an involuntary increase in call amplitude in response to masking ambient noise, represents one of the most efficient mechanisms for optimizing the signal-to-noise ratio.^6^^,^^7^^,^^8^^,^^9^^,^^10^ This effect is often accompanied by several other vocal changes, such as an increase in call frequency or an increase in signal density, i.e., the production of longer calls and/or increased repetition of call syllables.^10^^,^^11^^,^^12^^,^^13^ The latter increases the probability that a transmitted message will be received by another individual. These mechanisms elicited during the presence of masking noise are well known and occur across vertebrates from fish to birds to mammals, including humans.^5^^,^^9^^,^^14^^,^^15^^,^^16^

In addition to enhancing signal transmission in noisy environments, there is another vocal control mechanism that prevents vocal output during noisy events and induces animals to vocalize during periods when there is little or no acoustic disturbance. Previous studies showed that monkeys are able to time their vocalizations to silent epochs when time windows of ambient noise were predictable to avoid call transmissions with a low signal-to-noise ratio^17^ and to stop sequences of calls immediately after acoustic perturbation.^18^^,^^19^ Recently, we showed that marmosets are able to interrupt ongoing vocalizations directly after noise onset indicating that marmosets can avoid calling in noise on a rapid timescale.^20^ However, this behavior was very rare and happened only in 0.3%–7.7% of all cases (depending on the monkey). While the control mechanisms underlying vocal adjustments in response to environmental noise are already well studied, the mechanisms underlying noise avoidance strategies are still largely unclear. For example, it is not yet clear whether the ability to time and interrupt vocalizations in response to environmental noise is based on reflexive and/or adaptive processes.

Using a neuroethological approach, we systematically perturbed ongoing vocalizations of marmoset monkeys to investigate the nature of the strategies underlying the exhibited changes in vocal behavior and to decipher, why call interruption occurred only very rarely in our previous study.^20^ To do so, we perturbed ongoing vocalizations with noise presented at different time points and detected changes in vocal behavior that supported both reflexive and adaptive behavior in response to noise perturbation. Marmosets canceled their calls immediately after noise onset. Hereby, monkeys are more likely to cancel calling toward the expected end of calls rather than at the beginning of the vocalization suggesting that the rare occurrence of this behavior in an earlier study might be explained by the short noise onset latencies used.^20^ This behavior started during the first perturbed call, indicating a reflexive behavior in response to noise perturbation. In contrast, the reduction in number of syllables persisted beyond noise perturbation, indicating adaptive behavior in response to perturbing noise. Using machine learning techniques based on call parameters, we were able to show that even after the end of noise perturbation, some of the calls produced were initially planned by the monkeys as double phees but ended up being uttered with only one syllable. These findings suggest that marmoset monkeys use two different strategies to avoid noise perturbation during vocal behavior: a rapid one driven by reflexive responses and a more flexible, later occurring, and longer lasting adaptive one.

## Results

### Marmoset monkeys are able to change their vocal behavior in response to perturbing noise playback

**Figure 1:**
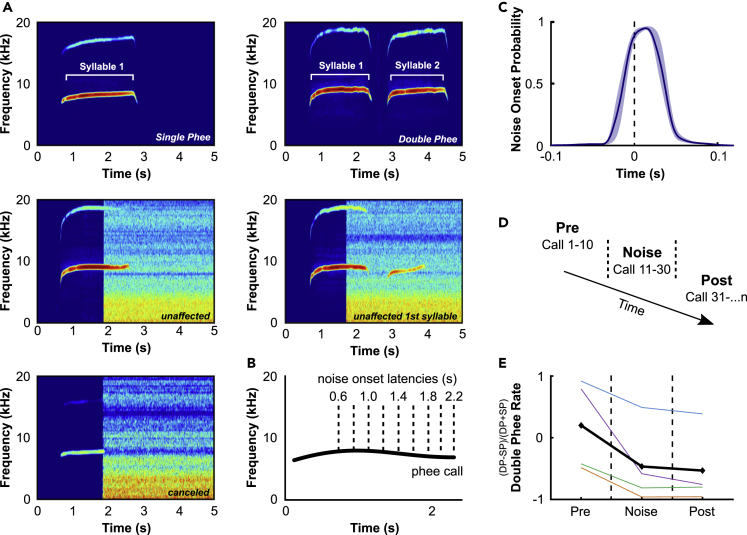
Marmoset monkeys are able to change their vocal behavior in response to perturbing noise playback (A) Exemplar spectrograms of single and double phees without and with noise perturbation. Unaffected: Calls with durations being unaffected by noise perturbation; Canceled: calls being canceled after noise onset. (B) Schematic of a phee call and the different used noise onset latencies indicated by the dashed lines. (C) Pooled noise onset probability normalized to set latencies (dashed line) ± SEM. (D) Schematic of the experimental procedure: pre-phase consisted of the first 10 calls without noise perturbation. Noise-phase consisted of the following 20 calls which were perturbed by noise. All subsequent calls (post) were again unperturbed. Arrow indicates the temporal trajectory of each session. (E) Double phee rate per experimental phase. 1: only double phees (DP) were emitted, −1: only single phees (SP) were produced. Different colors indicate different monkeys. The average is displayed in black. Noise-phase is indicated by dashed lines.

We measured the vocal behavior of marmoset monkeys (*Callithrix jacchus*, n = 4), while separated in a soundproofed chamber, with and without acoustic perturbation. In this setting, marmoset monkeys predominantly produced phee vocalizations.^10^^,^^20^ In total, we recorded 7945 phees (5843 single and 2102 double phees, Figure 1A). The first 10 calls of each session were not perturbed (pre-phase) and served as a control for each recording day (Figure 1D). The next 20 calls were perturbed in a precise manner if they exceeded a certain duration (noise-phase, Figures 1B and 1C). The rest of the calls produced after the noise-phase were again not perturbed (post-phase). With this experimental design, we were able to investigate changes of vocal behavior in response to perturbing noise as well as long-lasting, adaptive vocal changes after noise perturbation. We first investigated if the ratio of single to double phees was affected by noise perturbation. We found that marmoset monkeys emitted significantly more single phees in the noise- and post-phase than in the pre-phase (likelihood ratio [LR] test, χ^2^(2) = 687.781, p < 0.0001, Figure 1E). This indicated that the monkeys changed their vocal behavior in response to noise perturbation by producing fewer double phees, consistent with our previous findings.^20^

### The extent of phee duration changes is dependent on noise onset

**Figure 2:**
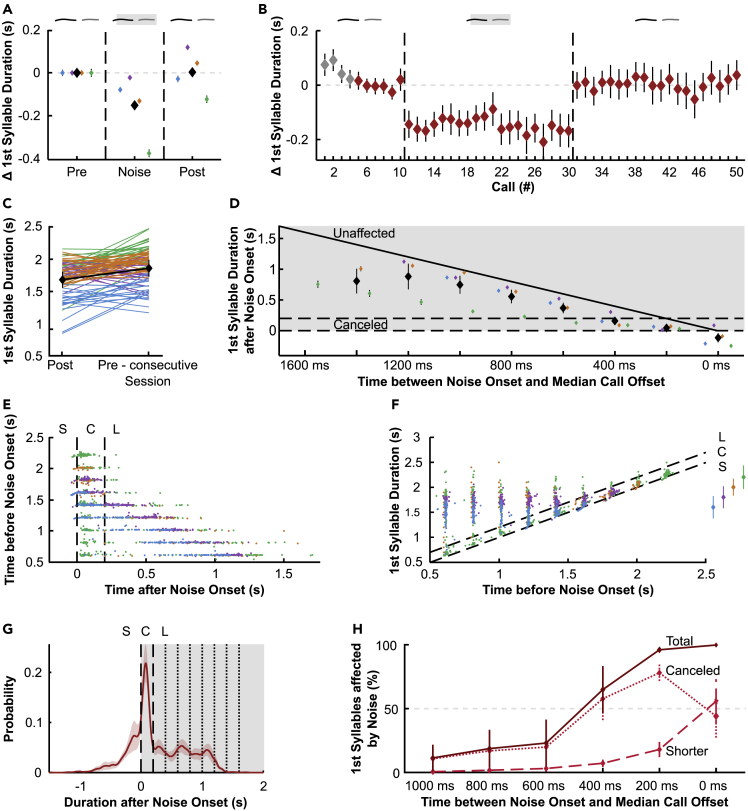
The extent of phee duration changes is dependent on noise onset Pooled Δ first syllable durations ± SEM in (A) noise- and post-phases per monkey (monkeys’ average displayed in black) normalized to pre data (gray dashed line) in (B) pooled call by call. The first 4 calls (indicated in gray) were discarded for a more stable baseline (see Methods for detail). (C) Average first syllable duration ± SEM of every 10 last calls per session (post) and first 10 calls of the consecutive session (pre-consecutive session), respectively. (D) Pooled Δ first syllable duration ± SEM after noise onset over time between noise onset and median call offset. Dashed lines show canceled calls (directly affected by noise). Angular line shows the values for unaffected calls. Noise perturbation is shaded in gray. (E) Call duration before noise onset as a function of call duration after noise onset. Dashed lines indicate canceled calls. S: Calls which were shorter than the given noise onset latency (calls ended before noise perturbation). C: Canceled calls (calls ended <200 ms after noise onset). L: Calls which ended >200 ms after noise onset. (F) Call duration as a function of call duration before noise onset. Inset on the right: median call duration for each monkey ± SE. Dashed lines indicate canceled calls. (G) Average first syllable call duration after noise onset ± SEM. Dashed lines indicate canceled calls. Dotted lines indicate noise onset latencies. Noise perturbation is shaded in gray. (H) Proportion of first syllables affected by noise perturbation against time between noise onset and median call offset ± SEM. Different colors indicate individual monkeys in (A), (C), (D), (E), and (F). See also Figure S1.

As a next step, we investigated the general effect of noise perturbation on call durations of first phee syllables (i.e., single phees and the first syllables of double phees) independently of the exact noise onset latency after call onset. We found a significant difference in call duration between the three experimental phases (linear mixed model [LMM], F(24,322) = 185.006, p < 0.0001, n: pre = 681, noise = 2023, post = 1624). Overall, call durations were significantly shorter during noise perturbation than during pre-phase (approximately 0.15 s); vocalizations produced in the post-phase did not show any differences to calls in the pre-phase (post-hoc Tukey: Pre vs. noise & noise vs. post p < 0.0001, pre vs. post p = 0.910, Figure 2A). However, the change in call duration across phases did not occur systematically across monkeys. The two female monkeys (orange and purple) showed an increase in first syllable duration in the post-phase compared to the pre-phase (p < 0.0001). In contrast, one of the males (green) showed a significant shortening of the first syllable when comparing the pre- and post-phases (p < 0.0001); the other male (blue) showed no differences in the length of the first syllable between the post- and pre-phases (p = 0.505).

To test how fast the monkeys modulated phee duration in response to noise perturbation, we measured the duration of the first phee syllables with respect to when they were uttered within the session. We found that across sessions, the first call perturbed with noise showed a significant decrease in call duration compared to the last call of the pre-phase, indicating an immediate response to noise perturbation (session calls 10 and 11; paired t-test, t(113) = 4.979, p < 0.0001, n = 117 pre-noise pairs). Similarly, the first unperturbed call after the noise-phase was longer than the last perturbed call (session calls 30 and 31; paired t-test, t(91) = 4.240, p < 0.0001, n = 98 noise-post pairs, Figure 2B). Furthermore, we wanted to know how persistent and long-lasting the change in call duration was. Therefore, we compared the duration of the last 10 calls within the post-phase with the duration of the first 10 calls of the pre-phase of the next day’s session. We found that the duration of first phee syllables was significantly greater than the duration of the last calls of the previous session (paired t-test, t(89) = 6.534, p < 0.0001, n = 90 post-pre pairs, Figure 2C). Next, we investigated whether call duration was dependent on the onset of noise perturbation related to call offset. During noise-phases, we perturbed calls with noise starting 0.6–2.2 s after call onset (see Methods for details). Because the monkeys exhibited different median call durations, we normalized noise onset latencies (which were initially set by call onset) to the time between noise onset and median call offset. For example, monkey H had a median first phee syllable duration of 1.6 s and monkey W of 2.2 s. Therefore, a noise onset time of 1.6 s resulted in 0 ms time between noise onset and median call offset for monkey H and 600 ms for monkey W. We assumed that if the monkeys perfectly responded to noise perturbation, they would routinely stop their vocalizations directly after perturbation onset (<200 ms after noise onset, horizontal dashed lines, Figure 2D). Alternatively, if the call durations were unaffected by the perturbing noise, they would still be similar to their regular median call duration as indicated by the angular line. However, we found that the monkeys did not purely show one or the other of these assumed behaviors. Their call durations showed both a significant difference from perfect compensation to noise perturbation (z-test, z(2596) = 34.171, p < 0.0001), as well as from fully unaffected call durations (z-test, z(2596) = 52.2, p < 0.0001), indicating a more complex vocal behavior in response to different noise onset times.

Consequently, we took a closer look at call duration distributions related to the different noise onset times. Particularly for the short noise onset latencies (time before noise onset), we found two distinct clusters for call duration after noise onset (Figure 2E). These two clusters were also evident when considering call durations in general (Figure 2E). In addition, call durations during noise perturbations were shorter than median call durations (Figure 2F). When analyzing call duration probabilities, we found a multimodal distribution for the pooled data including all used noise onset latencies (Figure 2G) as well as for the individual animals for most noise onset conditions (Figure S1), indicating that calls were either canceled directly after noise onset (call offset <200 ms after noise onset) or were unaffected by noise onset. Therefore, we divided call durations into three groups with respect to the relationship between call offset and noise onset: “shorter” for calls ending prior to noise onset, “canceled” for calls ending within 200 ms after noise onset, and “longer” for vocalizations that did not appear to be directly affected by noise onset, i.e., calls that did not end within the first 200 ms after noise onset. We found that the occurrence of these three durations showed significant differences within noise onset conditions (LR test, χ^2^(10) = 1984.646, p < 0.0001, Figure 2H). Canceled and shorter calls occurred predominantly in noise onset conditions that were close to the median call offset (400, 200, and 0 ms before median call offset). The presence of these call duration groups decreased rapidly with increasing time between noise onset and median call offset indicating that the monkeys have more control over call duration at the end of their vocalizations. Our results suggest that marmoset monkeys are capable of adaptively modifying the duration of ongoing phee vocalizations when facing perturbing noise.

### Second syllable duration changes due to noise perturbation

**Figure 3:**
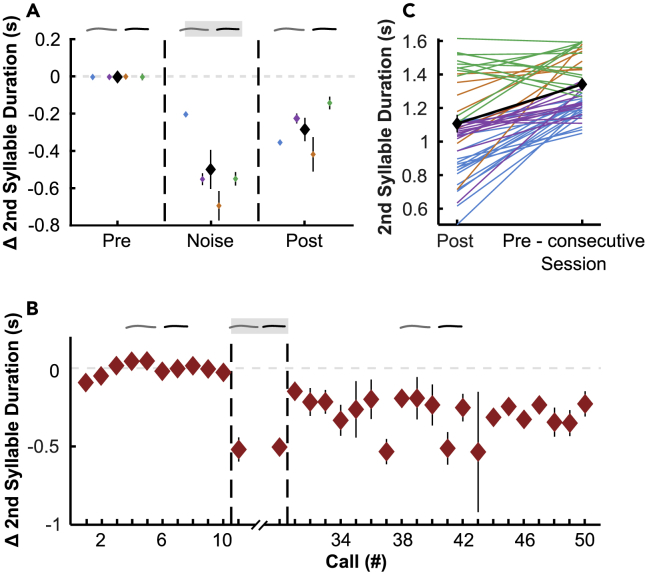
Second syllable duration changes due to noise perturbation Pooled Δ second syllable durations ± SEM in (A) noise- and post-phases per monkey normalized to pre data (gray dashed line) in (B) pooled call by call. (C) Average first syllable duration ± SEM of every 10 last calls per session (post) and first 10 calls of the consecutive session (pre-consecutive session), respectively. Different colors indicate different monkeys. The average is displayed in black. Noise-phase is indicated by dashed vertical lines. See also Figure S2.

Next, we investigated how the duration of second syllables of double phees, which were fully perturbed by noise, was affected. We found significant differences in second syllable durations between experimental phases (LMM, F(32,095) = 120.719, p < 0.0001, n: pre = 675, noise = 417, post = 764). Similar to call duration distributions of the first phee syllables, call durations were shorter during noise perturbation than in the pre-phase (0.48 s shorter than in the pre-phase). Interestingly, this effect was much stronger than for first syllable durations. Moreover, after noise perturbation (post-phase), durations of the second syllables were significantly shorter than in the pre-phase (0.28 s; post-hoc Tukey all comparisons p < 0.0001, Figure 3A). This means that in the rare cases where a second syllable was produced after noise perturbation, the duration of these calls was significantly shorter. As a next step, we wanted to test how fast the monkeys modulated the duration of the second syllables in response to noise perturbation. Therefore, we measured the duration of the second phee syllables as a function of when they were uttered within the session. As with the first syllables, we found that across all sessions, the first call perturbed with noise showed a significant decrease in call duration compared to the last call of the pre-phase, indicating an immediate response to noise perturbation (session calls 10 and 11; paired t-test, t(52) = 7.562, p < 0.0001, n = 53 pre-noise pairs). This effect could be found in all four monkeys (Figure S2). Similarly, the first unperturbed call after the noise-phase was again longer than the last perturbed call (session calls 30 and 31; paired t-test, t(35) = 3.708, p < 0.0001, n = 36 noise-post pairs, Figure 3B). Additionally, we also wanted to know how persistent and permanent the change in call duration was for the second phee syllables. Therefore, we also compared the duration of the last 10 calls in the post-phase with the duration of the first 10 calls in the pre-phase of the next day’s session. We found that the duration of the second phee syllables was significantly longer than the duration of the last calls of the previous session (paired t-test, t(47) = 6.856, p < 0.0001, n = 48 post-pre pairs, Figure 3C).

### Accuracy of single phee prediction for single phees produced during different experimental phases

**Figure 4:**
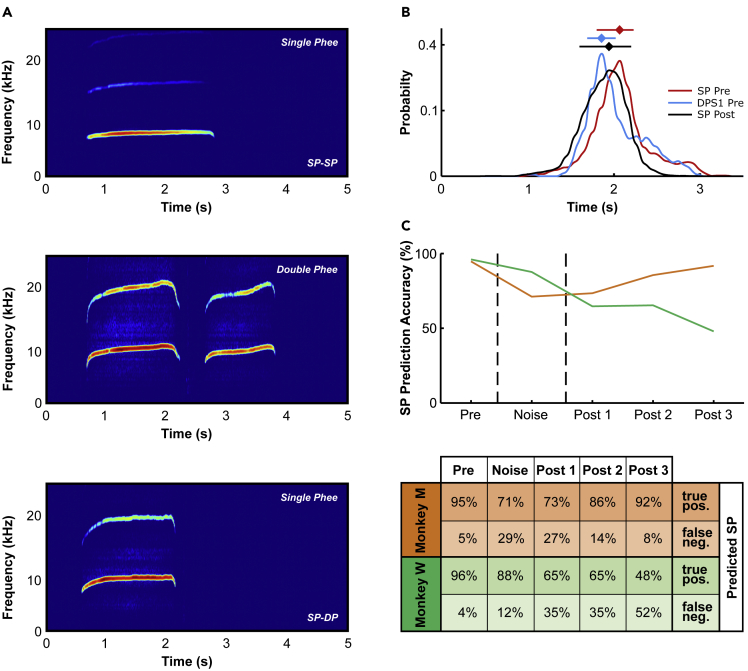
Single phee prediction accuracies for single phees produced during different experimental phases (A) Exemplar spectrograms of a single phee that was classified as a single phee, a double phee, and a single phee that was classified as the first syllable of a double phee. (B) Duration distributions of single phees (SP Pre) and first syllables of double phees (DPS1 Pre) in the pre-phase as well as single phee durations (SP Post) in the post-phase (monkeys M & W). Horizontal lines indicate full widths at half maximum and diamonds the positions of the maxima of the respective duration distributions. (C) Pre = first 10 calls, Noise = next 20 calls, Post = subsequent calls in groups of 20. Different colors indicate different monkeys. Noise-phase is indicated by dashed lines. See also Table S1.

Nevertheless, all monkeys barely produced double phees during the noise-phase. Does this indicate that monkeys switch their vocal behavior from double phees to single phees or that they cancel double phees after the first syllable? This is an important point because it allows us to determine the level of marmoset vocal plasticity with regard to vocal motor control. In the first case, marmosets would be able to adaptively change call initiation in response to perturbing noise; in the second case, they would be able to adaptively change a pre-determined behavior. This question was driven by our observation that while single phees were relatively stable in duration, single phees in the noise- and post-phase often had the same temporal structure and duration as the first syllable of a double phee (Figure 4A). Furthermore, the duration distribution of single phees in the post-phase encompassed the duration distribution of single phees and the first syllables of double phees (Figure 4B). Therefore, we hypothesized that some of the single phees in the noise- and post-phase are first syllables of double phees whose second syllable have been canceled after the production of the first syllable. To test whether single phees during noise- and post-phases were true single phees or the first syllable of double phees, we trained a machine learning classification model (medium support vector machine with 25% holdout) with several call features (for details see Table S1) of single and double phees produced in the pre-phase. Two monkeys produced only a few single phees and were, therefore, excluded from the analysis. For the other two monkeys, we obtained a predictive power of 95% and 96%, respectively, for the correct classification of single phees during the pre-phase, indicating that the classification model worked as expected. We then tested the model’s predictive power for single phees in the noise- and post-phases. We argue that if a fraction of produced single phees are first syllables of double phees that have been canceled, the predictive power of our classification model should drop significantly. Indeed, the predictive power was significantly decreased for monkey W in the noise- and post-phases (LR test, χ^2^(4) = 26.158, p < 0.0001; Figure 4C). Furthermore, for monkey M, we found a similar effect, with a decrease in predictive power in the noise- and first post-phase (LR test, χ^2^(4) = 9.474, p = 0.0503; Figure 4C). While the predictive power was also significantly lower in the post-phase for monkey W, it recovered for monkey M in the post-phase for single phees and increased during the post-phase in monkey W. These results provide the first evidence that marmoset monkeys can change the number of phee syllables after call onset.

## Discussion

In the current study, we showed that marmoset monkeys exhibit adaptive and reflexive noise-dependent changes in acoustic call structure. They produced fewer phee syllables while vocalizing in noise. This behavior remained after noise presentation, indicating an adaptive behavior. Furthermore, marmosets decreased their call durations as a direct response to noise perturbation during ongoing vocalizations in a reflexive manner. After periods of noise perturbation, calls were uttered immediately with durations similar to those being exhibited prior to noise perturbation. In double phees, this effect could be observed for the first syllable, which was perturbed by noise after syllable onset, as well as for the second syllable, which was perturbed by noise from beginning to end.

Vocal changes as direct responses to perturbing noise, such as involuntary increases in call amplitude (Lombard effect) and associated changes, including increases in call frequency and call duration, have been extensively studied in recent decades across vertebrate species such as birds, reptiles, and mammals, including cetaceans, bats, and primates.^13^^,^^14^^,^^15^^,^^16^^,^^17^^,^^19^^,^^21^^,^^22^^,^^23^^,^^24^ The observed changes resulted either in an increase in signal-to-noise ratio, such as the Lombard effect, and an increase in call frequency or an increase in signal density, such as in the production of longer calls and/or increased repetition of call syllables, both of which resulted in a potential increase in signal transmission probability.

In contrast, noise avoidance strategies, i.e., suppressing vocalizations during periods of elevated ambient noise levels, have received little attention so far. Monkeys have been shown to avoid calling in noisy environments and time their calls to silent epochs^17^ and stop call sequences immediately after noise onset.^18^^,^^19^ Marmosets are even able to interrupt ongoing vocalizations directly after the onset of perturbing noise.^20^ However, the underlying mechanisms remain largely unclear.

In our study, marmosets were able to cancel their calls immediately after noise onset. This behavior started during the first perturbed call, indicating a reflexive behavioral reaction to noise perturbation. Here, it was easier for the animals to cancel calls toward the expected end of the call rather than at the beginning of the vocalization. These results suggest underlying neural mechanisms that might inhibit the interruption of vocalizations at the beginning of the pattern.^25^ This would explain the low occurrence of interrupted calls (0.3%–7.7%) in our earlier study, in which we perturbed vocalizations immediately after call onset.^20^ These findings further support the hypothesis that call patterns are more stable at the beginning of a vocalization and can be modulated toward the end. Neurophysiological studies are now needed to reveal the underlying neural mechanisms that explain more stable calling behavior in the initial part of a vocalization and a release of the above-mentioned inhibition toward the end of calls.

Interestingly, the monkeys seem to have different strategies to deal with noise when uttering first and second phee syllables. For the second phee syllables, all monkeys showed the same strategy and continued to utter these syllables with shorter duration even after noise perturbation ended. For first phee syllables, however, there was not such a systematic change in vocal behavior. While one monkey remained shorter even after noise perturbing ended, as with the second syllables, the two female monkeys showed a significant increase in call duration even after noise perturbation ended.

Regarding call sequence structure, our study shows, similar to previous studies,^18^^,^^19^ that the occurrence of double phee calls significantly decreased during noise perturbation, indicating that marmosets terminated the sequence directly after perturbation onset within the first phee syllable, irrespective of whether they canceled the first syllable. Moreover, we revealed that this behavior persisted after noise perturbation ended, indicating that the monkeys exhibited adaptive changes in their vocal behavior. Interestingly, our machine learning classification model based on call parameters that were not directly affected by the perturbing noise gave the first evidence that some uttered single phees were planned to be doubles phees that were canceled after the first syllable during noise- and post-phases. These findings further show that marmosets have direct control over their vocal output and are capable of modulating ongoing vocalizations in a rapid and direct way.

### Limitations of the study

We observed sex-related differences in one aspect of the calling behavior studied in response to noise perturbation. However, to verify whether these are truly sex-specific differences, the sample size used in the present study is too small. Future work with larger number of animals will have to show whether sex-specific differences in noise avoidance strategies exist in marmoset vocal behavior.

## STAR★Methods

### Key recources table

**Table:** 

REAGENT or RESOURCE	SOURCE	IDENTIFIER
**Experimental models: Organisms/Strains**		

*Callithrix jacchus*	German Primate Center, Göttingen, Germany, and Werner Reichardt Centre for Integrative Neuroscience, University of Tübingen, Germany	N/A

**Software and algorithms**		

MATLAB	MathWorks	R2021b
OpenEx	Tucker-Davis Technologies	N/A
JMP	SAS Institute	version 16
SASLab Pro	Avisoft Bioacoustics	version 5.2.09

### Resource availability

#### Lead contact

Further information and requests for resources should be directed to and will be fulfilled by the lead contact, Steffen R. Hage (steffen.hage@uni-tuebingen.de).

#### Materials availabilities

This study did not generate new unique reagents.

### Experimental model and subject details

#### Animals

Four adult marmoset monkeys (*Callithrix jacchus*, two females aged 6 and 7 years and two males aged 3 and 6 years, respectively, at the beginning of the experiments) were used in this study. The monkeys were usually kept in different sex pairs and were all born in captivity. The animals had *ad libitum* access to water and were fed on a restricted food protocol including a daily basis of commercial pellets, fruits, vegetables, mealworms, and locusts. Additional treats, such as marshmallows or grapes, were used as positive reinforcements to transfer animals from their home cage to the experimental cage. Environmental conditions in the animal husbandry were maintained at a temperature of 26°C, 40–60% relative humidity, and a 12 h:12 h day/night cycle, including periods of twilight in the morning and evening. All animal handling procedures were in accordance with the guidelines for animal experimentation and authorized by the national authority, the Regierungspräsidium Tübingen.

### Method details

#### Experimental setup and procedure

The vocal behavior of four animals in response to noise playback, which was initiated with different latencies relative to the vocal onset, was recorded in a soundproof chamber. The tested animal was transferred into a recording cage (0.6 × 0.6 × 0.8 m) with *ad libitum* access to water and food. In this behavioral setup, marmoset monkeys predominantly produce phee calls (long distance contact calls) spontaneously.^10^^,^^20^^,^^26^^,^^27^ The vocal behavior of each individual monkey was recorded in daily sessions ranging between 30 and 150 min in duration. Data was collected in sessions at various times during the day between 11 am and 5 pm. Recordings were performed for 21–39 days for each individual animal (mean: 29 ± 7 days). The monkey’s behavior was constantly monitored and observed using a webcam (Logitech, Switzerland) placed on top of the cage and recorded with standard software (Synapse Tucker-Davis Technologies, U.S.A.). The vocal behavior was recorded using a microphone (MKH 8020 microphone with MZX 8000 preamplifier, Sennheiser, Germany) and digitized using an A/D interface (RX8, Tucker-David Technologies, U.S.A.). A custom-written program (OpenEX and Synapse, Tucker-Davis Technologies, U.S.A.) running on a workstation (WS-X in combination with an RZ6D multi I/O processor, Tucker-Davis Technologies, U.S.A.) recorded the emitted vocalizations and monitored them in real-time. Our vocal detector automatically detected calls in real-time through online calculation of several acoustic parameters, such as call intensity, minimum call intensity duration, call frequency, and several spectral features.

##### Experimental design

To investigate the effect of noise perturbation during ongoing vocalizations, we partitioned each session into three phases. The animal’s first 10 phee calls uttered during a session were kept unperturbed and used as a control for the subsequent performance (pre-phase). For the next 20 phee calls that were longer than the set noise onset latency (between 0.6 s and 2.2 s; see below), we played broadband noise bursts (0.1–60 kHz) with an intensity of 80 dB SPL via a loudspeaker (MF1 Multi-Field Magnetic Speakers, Tucker-Davis Technologies, U.S.A.) positioned on top of the cage (noise-phase). All phee calls following the noise-phase were again unperturbed (post-phase) (Figures 1A, 1B and 1D).

Noise bursts had a duration of 4 s (including 10 ms rise times) to ensure noise perturbation throughout the first syllable after noise onset as well as the entire potential second phee syllable. In total, we used up to nine different noise latencies depending on the monkeys' median call duration. The shortest latency for noise onset after call onset was 0.6 s. The longest latency was defined as the individual monkey’s median call duration, which was 2.2 ± 0.234 s (SE) for monkey W, 2.0 ± 0.170s for monkey M, 1.8 ± 0.219 s for monkey S, and 1.6 ± 0.223 s for monkey H. Additionally, we used noise latencies in steps of 200 ms between the shortest latency and the monkeys’ median call duration resulting in nine noise latencies for monkey W (0.6–2.2 s in 200 ms steps), eight latencies for monkey M (0.6–2.0 s), seven latencies for monkey S (0.6–1.8 s), and six latencies for monkey H (0.6–1.6 s). All noise latencies were tested for at least three sessions in a block design per individual monkey. For noise onset determination, we used our call detector and added the corresponding noise latency used in the appropriate session. Figure 1C shows the distribution of noise onsets normalized to the set latencies with a mean shift of 13 ± 0.06 ms (SEM), indicating precise signal detection.

##### Data analysis

Vocal onsets and offsets were manually flagged, as well as noise onset times, using standard software (Avisoft-SASLab Pro, Germany). Call duration was calculated as the difference between the beginning and end of the vocalization. The spectrograms were calculated using a 512-point FFT Hanning window (256 samples), and 128-sample overlap resulting in a frequency resolution of 191 Hz and temporal resolution of 2.6 ms.

##### Data normalization

All used call values were normalized by subtracting the mean of the last six calls produced in the pre-phase per respective session. We used the last six calls instead off all calls emitted during pre-phase to get a stable baseline since the monkeys needed some time to settle down (comparison between first 3 and last 3 calls of the 6 calls used for the new baseline, t(20) = 1.527, p = 0.141).

##### Single/double phee classifier

We used a medium gaussian support vector machine (SVM) classifier with 25% holdout validation (standard Matlab classification learner app; R2020a MathWorks, U.S.A.) to evaluate the predictive power of certain call features of the first phee syllable for whether they are single phees or the first syllable of a double phee.^28^ Twenty-four custom call features (Table S1) were defined and computed in order to predict the phee syllable including entropy, bandwidth, maximum and peak frequency at specific time points (20-, 250- and 500 ms after call onset), and the slope between peak and maximum frequencies between 20 ms and 500 ms. We only used call features that could be calculated from the first 500 ms of the calls, since noise perturbation, which was capable of modifying call structure, started in some sessions at 600 ms after call onset.

### Quantification and statistical analysis

To evaluate a balanced single to double phee ratio among experimental phases, we performed a two-dimensional likelihood ratio test to examine whether observed frequencies differ between pre, noise, and post-phases. To understand variations in phee call first and second syllable durations, we constructed an LMM with experimental phase (pre, noise, post) as a nominal predictor variable. We further added monkey ID as a random factor to correct for variances in inter-individual differences. To evaluate if there were any differences in first and second syllable call duration between the last call in the pre-phase and first call in the noise-phase, as well as the last call in the noise-phase and first call in the post-phase, we performed paired t-tests. To test if there were any differences in first and second syllable call durations between the sessions’ last 10 calls (post) and the first 10 calls (pre) of the following session, we performed a paired t-test. To understand variations in phee call first syllable duration after noise onset, we used z-tests with subsequent Bonferroni correction to compare the measured distributions to either a perfect noise compensated distribution or a fully unaffected call length distribution. To evaluate balanced occurrences of phee durations among latency conditions, we performed a two-dimensional likelihood ratio test to examine whether observed frequencies of shorter, canceled, and longer calls differed between noise latencies. We also performed a two-dimensional likelihood ratio test to examine whether the number of manually labeled single phee calls differed from the count labeled by the classifier over the experimental phases (pre, noise, and post in groups of 20 calls). All statistical analyses were performed using JMP16 (SAS Institute, U.S.A.). In all performed tests, significance was tested at an alpha level of 0.05.

## Data Availability

• Data All data needed to evaluate the conclusions in the paper are present in the paper. Additional data related to this paper is available from the lead contact upon request.• Code This paper does not report original code.
