# Supplementary material for: Marmoset monkeys use different avoidance strategies to cope with ambient noise during vocal behavior
Source: iScience. 2023 Feb 16;26(3):106219. doi: 10.1016/j.isci.2023.106219 (PMC10006620; doi:10.1016/j.isci.2023.106219)
Supplement: Document S1. Figures S1 and S2 and Table S1 [file mmc1.pdf]

**iScience, Volume *26***

## Supplemental information

# Marmoset monkeys use different avoidance strategies to cope with ambient noise during vocal behavior

**Julia Löschner, Thomas Pomberger, and Steffen R. Hage**

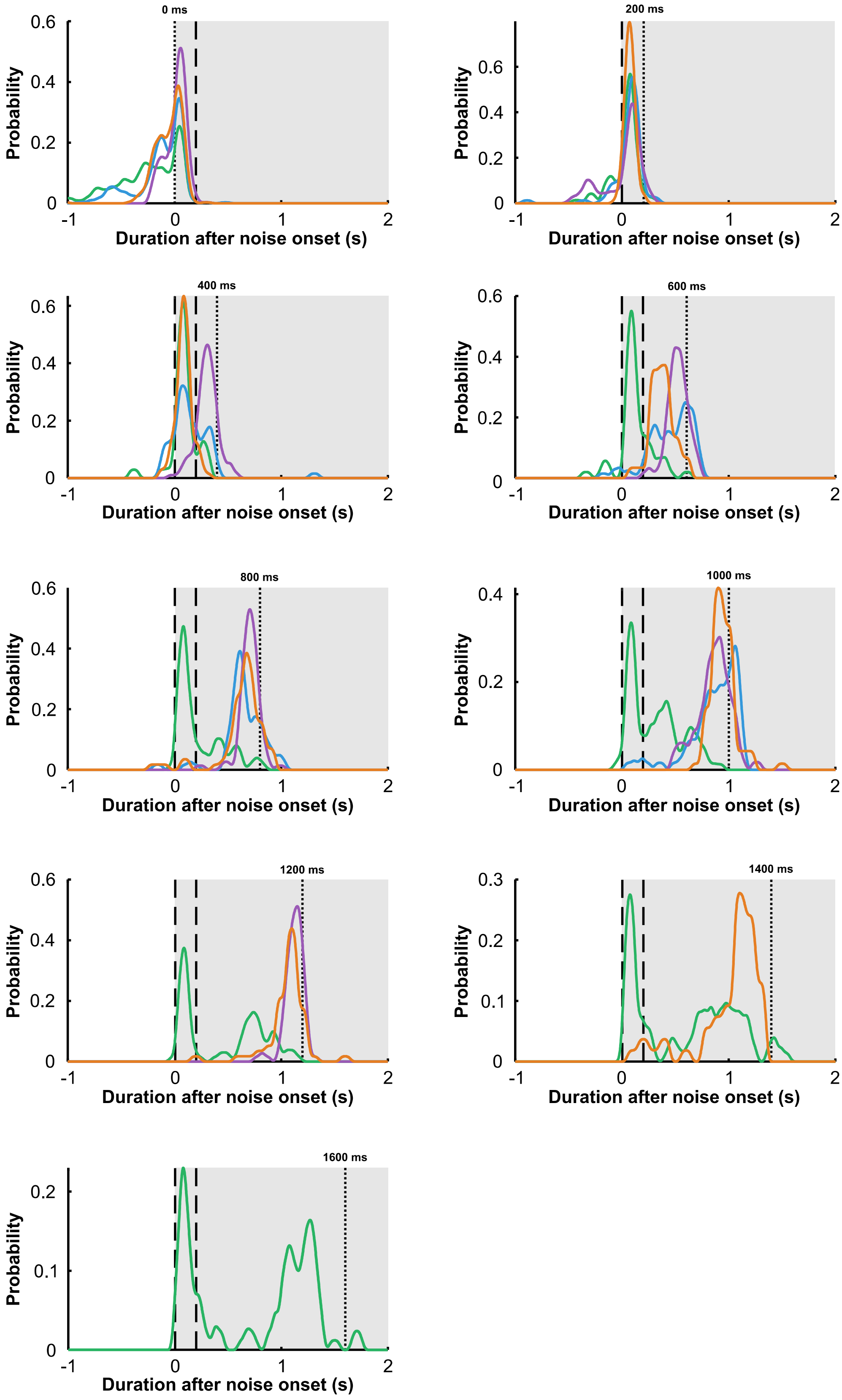


**Figure S1, Related to Figure 2:** Average first syllable call duration after noise onset per different noise onset latencies (dotted lines). Dashed lines indicate canceled calls. Noise perturbation is shaded in grey.

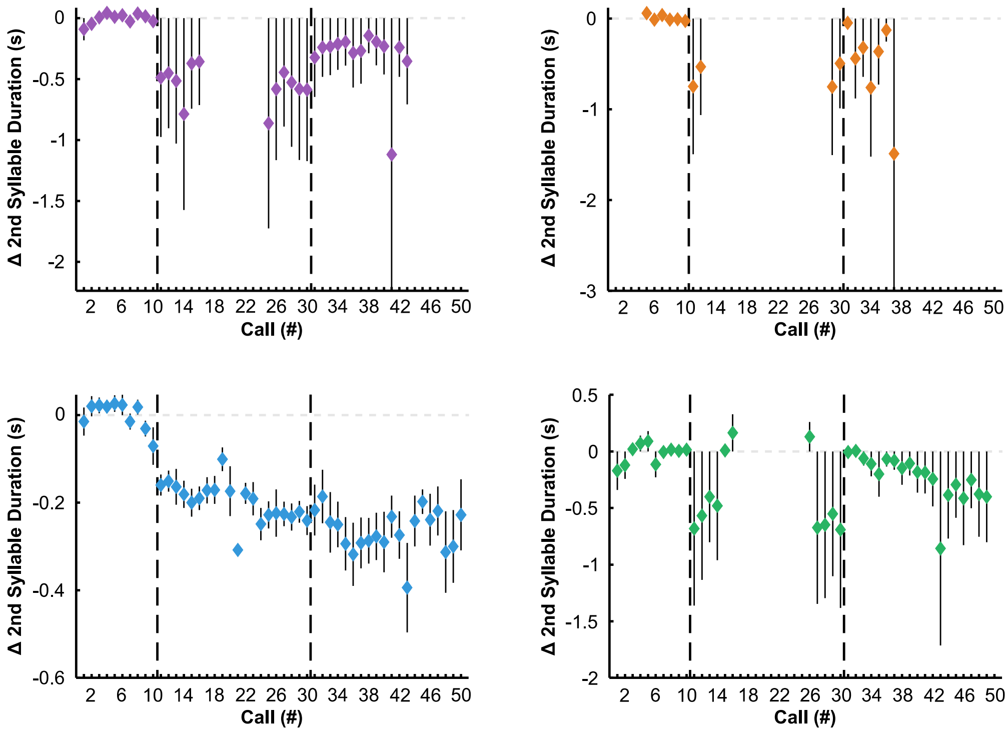


**Figure S2, Related to Figure 3:** Pooled Δ second syllable durations ± SEM call by call per individual monkey. Noise-phase is indicated by dashed lines.

**Table S1, Related to Figure 4:** Spectral features used for SVM classifier (definition according to Avisoft).

| Medium SVM Features | Explanation |
|---|---|
| Distance to maximum | The distance from start to the location of maximum amplitude. |
| Peak Frequency (20, 250, 500 ms) | Frequency of maximum power. |
| Max Frequency (20, 250, 500 ms) | Frequency where the magnitude of the spectrum first exceeds the specified threshold (referenced to the peak [maximum] amplitude) towards higher frequencies. |
| Bandwidth (20, 250, 50 ms) | Difference between maximum and minimum frequency. |
| Slope Peak Frequency | Difference between 500 and 20 ms peak frequency over time. |
| Slope Max Frequency | Difference between 500 and 20 ms maximum frequency over time. |
| Entropy (20, 250, 500 ms) | Quantifies spectral flatness (or tonality) of a sound. Ratio of the geometric mean to the arithmetic mean of the spectrum. This parameter is (theoretically) 0 in pure tone signals and 1 in random noise. |
| Quartiles 25%, 50%, 75% (20, 250, 500 ms) | Parameters characterize the distribution of the energy on the spectrum. The area below the spectrum will be integrated and divided into four equal parts. The three frequencies dividing the magnitude spectrum into four equal parts are called quartiles. Quartile 25% splits the lowest 25% of the spectrum from the highest 75%. Quartile 50% splits the spectrum in half. Quartile 75% splits the highest 25% of the spectrum from the lowest 75%. |
